# Selection Mosaic Exerted by Specialist and Generalist Herbivores on Chemical and Physical Defense of *Datura stramonium*

**DOI:** 10.1371/journal.pone.0102478

**Published:** 2014-07-22

**Authors:** Guillermo Castillo, Laura L. Cruz, Rosalinda Tapia-López, Eika Olmedo-Vicente, Diego Carmona, Ana Luisa Anaya-Lang, Juan Fornoni, Guadalupe Andraca-Gómez, Pedro L. Valverde, Juan Núñez-Farfán

**Affiliations:** 1 Departamento de Ecología Evolutiva, Instituto de Ecología, Universidad Nacional Autónoma de México, Mexico City, Distrito Federal, México; 2 Departamento de Ecología Funcional, Instituto de Ecología, Universidad Nacional Autónoma de México, Mexico City, Distrito Federal, México; 3 Departamento de Biología, Universidad Autónoma Metropolitana Iztapalapa, México City, Distrito Federal, México; College of Charleston, United States of America

## Abstract

Selection exerted by herbivores is a major force driving the evolution of plant defensive characters such as leaf trichomes or secondary metabolites. However, plant defense expression is highly variable among populations and identifying the sources of this variation remains a major challenge. Plant populations are often distributed across broad geographic ranges and are exposed to different herbivore communities, ranging from generalists (that feed on diverse plant species) to specialists (that feed on a restricted group of plants). We studied eight populations of the plant *Datura stramonium* usually eaten by specialist or generalist herbivores, in order to examine whether the pattern of phenotypic selection on secondary compounds (atropine and scopolamine) and a physical defense (trichome density) can explain geographic variation in these traits. Following co-evolutionary theory, we evaluated whether a more derived alkaloid (scopolamine) confers higher fitness benefits than its precursor (atropine), and whether this effect differs between specialist and generalist herbivores. Our results showed consistent directional selection in almost all populations and herbivores to reduce the concentration of atropine. The most derived alkaloid (scopolamine) was favored in only one of the populations, which is dominated by a generalist herbivore. In general, the patterns of selection support the existence of a selection mosaic and accounts for the positive correlation observed between atropine concentration and plant damage by herbivores recorded in previous studies.

## Introduction

Coevolution, the reciprocal evolutionary change between interacting species, has been considered a key process in the evolution of both plants and their natural enemies [1]–[4]. In particular, it has been used to explain the evolution of the great diversity of defensive traits in plants, such as trichomes, spines, resins, or secondary metabolites [5], [6]. This theory assumes that herbivores exert selective pressures on traits that reduce herbivore damage [4], [7]. Given that herbivory generally decreases plant fitness, natural selection is expected to favor high levels of these defensive traits [8]–[10].

Nonetheless, plant populations are often distributed along wide geographic areas, and are thus exposed to different herbivore communities, ranging from generalists (which feed upon a wide diversity of hosts), to specialists (which feed on a related group of species) [11]–[13]. It has been hypothesized that defensive traits are an effective barrier against generalist herbivores, because these herbivores can feed on alternative plants, (but see [14]). On the other hand, several studies have suggested that specialist herbivores have evolved mechanisms to overcome host defenses [6], [12], [15], [16]. Moreover, specialist herbivores may be able to identify their hosts based on defensive traits such as secondary metabolites, imposing negative selection on these traits [11], [17], [18]. Thus, along the distribution of a plant species, defensive traits such as trichomes and secondary metabolites may be under contrasting selective pressures arising from multiple interacting species [19]. Such spatially variable selection is expected to change the population mean of traits, promoting population differentiation in defensive traits [20]–[22]. Although there is much evidence of selection on plant defenses, there is less evidence regarding spatially variable selection by herbivores on defensive traits (see [23], [24]). Furthermore, few studies have explicitly evaluated whether specialist and generalist herbivores exert different selection pressures on defensive traits [12].

*Datura stramonium* (Solanaceae) is an ideal system for studying variable selection patterns acting on defensive traits at a geographic scale. It typically grows in disturbed and agricultural habitats in Mexico, Canada, and the United States [25]–[27]. Due to its wide distribution, *D. stramonium* is exposed to a wide variety of herbivores and diverse environmental conditions. Most Mexican populations of *D. stramonium* are attacked by the specialist herbivore *Lema daturaphila* [28]. However, there are populations where *L. daturaphila* is absent, and where the main herbivores are *Epitrix parvula*, (specialist herbivore of the Solanaceae family) [29], and the generalist *Sphenarium purpurascens* (Núñez-Farfán and Guillermo Castillo, pers. obs.). Specifically, *D. stramonium* features leaf trichomes and tropane alkaloids as defensive traits that prevent herbivory [16], [26]. Previous studies have documented that these traits can evolve by selection by herbivores [16], [30]. Atropine is the substrate alkaloid used to produce the derived and more toxic scopolamine [31]. Recently, Castillo et al. [32] found a positive geographic association between atropine concentration and leaf damage across 28 *D. stramonium* populations in central Mexico, suggesting that atropine may not be an effective deterrent against herbivory. However, it is unclear whether selection exerted by generalist and/or specialist herbivores of *D. stramonium* drive this pattern. Specialized herbivores are expected to promote a more intense coevolutionary dynamic with the host plant, as they are more likely to adapt to the host chemical and physical barriers, imposing strong selection, and promoting counter-resistance host response [33]–[35]. Thus, while atropine may be less effective against herbivores than scopolamine [36], the benefits of tropane alkaloids should be higher against generalist rather than specialist herbivores.

Here, we evaluated whether selection imposed by specialized and/or generalized herbivores on plant defenses matches the among-population variation in defensive traits of *D. stramonium* recorded in a previous study [32]. To do so, we performed phenotypic selection analyses to explore the mode and intensity of selection acting on chemical (atropine, scopolamine) and physical (leaf trichomes) defensive traits in eight populations of *D. stramonium* attacked mainly by generalist or specialist herbivores. Next, we explored whether higher concentration of the derived alkaloid (scopolamine) is associated with higher plant fitness benefits, and whether selection for this secondary compound is more intense against generalist than specialist herbivores.

## Methods

### Ethics Statement

No specific permissions were required to make observations and to collect plant material of *D. stramonium* in the locations sampled in this study, nor is this species endangered and protected by the Mexican Government.

### Study system

*Datura stramonium* L. (Solanaceae) is an annual herb commonly distributed in cultivated areas, roadsides and disturbed environments in Mexico, the United States, Canada, and Europe [16], [26], [28], [37], [38]. This species reproduces mainly by self-fertilization, and has limited pollen and seed dispersal [39]. Leaves of *D. stramonium* are consumed primarily by the specialist folivorous beetle *Lema daturaphila* [28], the oligophagous flea beetle *Epitrix parvula* (which consumes other members of the Solanaceae family) [29], and the generalist grasshopper *Sphenarium purpurascens* [28]. *Lema daturaphila* damage is characteristic, as both adults and larvae consume the leaf blade while avoiding the main vascular bundles (pers. obs.). *Epitrix parvula* damage consists of small holes on the leaves. Although damage can be severe, whole leaves are rarely totally consumed [28]. Damage exerted by *Sphenarium purpurascens* consists of round-to-ragged holes in the leaves, typically originating from the leaf margins. Although leaf damage can be complete, grasshoppers usually leave partially defoliated leaves (G. Castillo personal observation). Previous studies have found that leaf damage significantly reduces plant fitness [28], [40] and that leaf trichomes and tropane alkaloids are defensive traits against herbivores [16], [26], [30], [32], [41].

### Sampled Population

**Figure 1:**
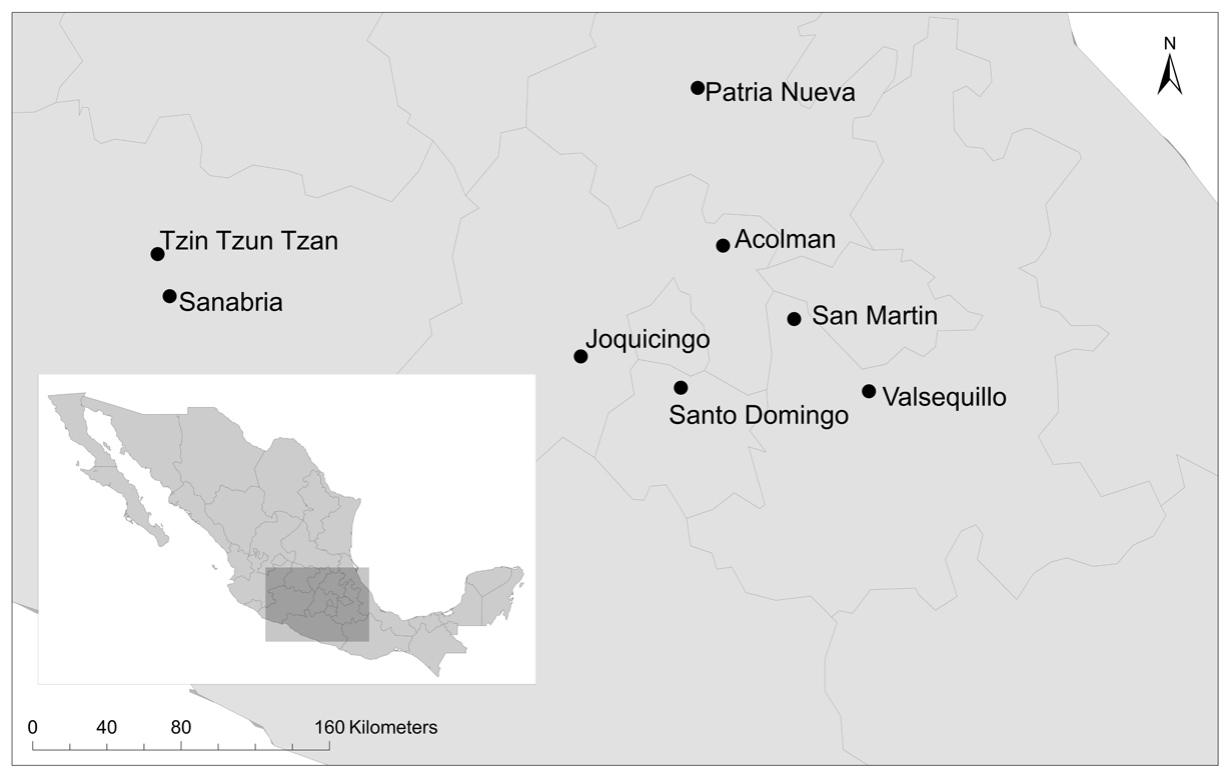
*Datura stramonium* populations sampled in Central Mexico (See Table S1).

From August-September 2011 we sampled eight natural populations of *D. stramonium* in central Mexico (Fig. 1). The sampled populations occurred within different plant communities (see Table S1). The linear distances between populations ranged from 20 to 300 km. In each population we sampled ±30 randomly selected individual plants. From each plant we collected a random sample of 20 leaves, and all the fruits produced. In addition, we recorded the predominant damage type caused by each herbivore feeding on *D. stramonium* in each population. The most frequent herbivore species at each population is listed in Table 1. Our field observations and leaf damage records during 3 years indicate that the predominant herbivores at each population remained stable throughout the 2010–2012 period.

**Table 1:** Selection differentials (*S*) of trichome density, atropine, and scopolamine concentration in eight populations of *Datura stramonium*.

Population	Main herbivore	Defensive trait	*S*	*SE*	*t*	*P*
Acolman (N = 31)	*Lema daturaphila*	Trichome density	0.116	0.192	0.604	0.55
		Atropine	**0.416**	**0.182**	**−2.284**	**0.029**
		Scopolamine	−0.089	0.197	−0.456	0.652
Patria Nueva (N = 30)	*Lema daturaphila*	Trichome density	−0.197	0.103	−1.905	0.067
		Atropine	−0.183	0.104	−1.753	0.09
		Scopolamine	**−0.269**	**0.097**	**−2.756**	**0.01**
Joquicingo (N = 31)	*Lema daturaphila*	Trichome density	−0.234	0.184	−1.277	0.211
		Atropine	−0.303	0.187	−1.62	0.116
		Scopolamine	−0.036	0.195	−0.186	0.854
San Martín (N = 29)	*Lema daturaphila*	Trichome density	0.205	0.151	1.36	0.186
		Atropine	**−0.331**	**0.142**	**−2.332**	**0.027**
		Scopolamine	−0.106	0.154	−0.685	0.499
Tzin Tzun Tzan (N = 30)	*Epitrix parvula*	Trichome density	**−0.347**	**0.099**	**−3.488**	**0.001**
		Atropine	−0.151	0.122	−1.233	0.228
		Scopolamine	0.107	0.128	0.837	0.41
Valsequillo (N = 33)	*Epitrix parvula*	Trichome density	−0.051	0.099	−0.552	0.605
		Atropine	**−0.255**	**0.088**	**−2.89**	**0.006**
		Scopolamine	−0.052	0.099	−0.533	0.598
Sanabria (N = 34)	*Sphenarium purpurascens*	Trichome density	−0.188	0.187	−1.004	0.324
		Atropine	−0.228	0.178	−1.281	0.209
		Scopolamine	−0.071	0.182	−0.39	0.699
Santo Domingo (N = 30)	*Sphenarium purpurascens*	Trichome density	−0.083	0.204	−0.409	0.686
		Atropine	**−0.392**	**0.191**	**−2.055**	**0.049**
		Scopolamine	**0.65**	**0.164**	**3.965**	**0.001**

Significant values appear in bold-type fonts. Standard error (*SE*) of estimates, *t*-test value, and probability (*P*) are provided.

### Trichome density and plant fitness

Trichome density was estimated as the total number of trichomes in an observation field of 2.5 mm^2^ located in the central basal region of the adaxial side of the leaf [26]. Average trichome density per plant was obtained from a sample of 20 randomly chosen, fully expanded leaves. The mean trichome density for each population was calculated for a sample of approximately 30 individuals. We used total fruit number as a proxy of plant fitness. Since *D. stramonium* is an annual selfing plant, fruit number is a good estimator of lifetime maternal fitness [8].

### Tropane alkaloid concentration

We used HPLC to quantify the concentration of atropine and scopolamine (two major alkaloids in *D. stramonium*) from a sample of 20 leaves per plant. The extraction method consisted of a series of acid-base reactions (see [32]). The samples were injected into a Waters Alliance 2695 HPLC device. We used a reverse-phase column (Discovery C-18 Supelco Analytical) at 30°C. The injection volume was 30 µL with a flow rate of 1 mL/min. The mobile phase was a solution of acetonitrile, methanol and a 30 mM phosphate buffer at a pH of 6.00 (9∶6.9∶84.1, v/v/v). The DAD detector used a wavelength of 210 nm. The curves obtained from each sample were compared against a standard solution of atropine and scopolamine (Sigma-Aldrich Laboratories, 1 mg/mL). The mean alkaloid concentration (mg/g) per population was estimated from a sample of ±30 plants.

### Data analysis

#### Among-population variation in plant defenses

Before conducting statistical analyses, we assessed among-population differences in defensive traits of the studied eight populations. A multivariate analysis of variance (MANOVA) was performed on leaf damage, trichome density, and concentrations of atropine and scopolamine. These analyses were equivalent to those published elsewhere (see [32]), but applied in this case to our eight selected populations. Our analyses detected significant differences in defensive traits among the eight populations of *D. stramonium* (Wilks' *λ* = 0.1477, *F*_28, 848.73_ = 30.697, *P*<0.0001). Univariate ANOVAs showed significant differences in trichome density (*F*_7, 238_ = 7.55, *P*<0.0001), atropine (*F*_7, 239_ = 2.96, *P* = 0.0053) and scopolamine concentration (*F*_7, 239_ = 4.10, *P* = 0.0003) (Fig. S1).

#### Phenotypic selection on defensive traits

Following the Lande and Arnold approach [42], we used multivariate selection analyses to estimate the magnitude and direction of linear and non-linear selection acting on defensive traits for each population. Standardized partial linear selection gradients (*β*) were obtained by fitting a linear regression that considered relative plant fitness as the response variable and all three defensive traits as predictor variables. Because our sample size precluded the estimation of reliable non-linear selection gradients, only directional selection gradients are presented here. Defensive traits were standardized (*µ* = 0, *σ^2^* = 1) and fitness was relativized for each population prior to the analyses. Regression analyses were performed using the function *lm* in R 3.0.2 [43].

Before conducting selection analyses, correlations between predictor variables were examined within each population to avoid strong multicollinearity in subsequent analyses. These analyses revealed that concentrations of atropine and scopolamine were positively correlated in six out of eight populations (Table S2). Only in two populations scopolamine was positively correlated with trichome density (Table S2). All other correlations between defensive traits were non-significant (Table S2). Therefore, we estimated selection differentials, as the slope of the univariate regression of population relative fitness on standardized traits [44]. These estimates measure changes in the distribution of a trait due to direct and indirect selection, when traits are correlated.

#### Differential selection among herbivore species

To assess whether patterns of selection on defensive traits are consistent among populations and herbivores, we estimated effect sizes for each differential and selection gradient. Effect sizes were used to compare estimates of phenotypic selection corresponding to populations consumed by different herbivore species [45]. Because the differentials and selection gradients were obtained from regression models with the same covariance structure, slopes are reliable metrics to estimate effect sizes [46]. Effect sizes were estimated using the slopes and their corresponding variances (estimated as: *V_β_ = SE_β_^2^*) [47] to weight each of them by level of certainty. In order to account for between-population variation, we applied a random-effect model following an Omnibus Test (*Q_m_*) [45]. We concluded that, when confidence intervals around mean effect size did not overlap with zero, a particular species of herbivore exerted a significant effect on the pattern of selection of a focal defensive trait. We used *metafor* [48] from R package to perform the analyses.

## Results

### Phenotypic selection on defensive traits

Multiple regression analyses revealed significant directional selection acting on defensive traits in five out of the eight studied populations (see Table 1). Trichome density was negatively selected in the Tzin Tzun Tzan population, and positively selected in the San Martín and Santo Domingo populations. Atropine concentration was negatively selected in the Acolman, San Martin and Valsequillo populations, whereas scopolamine concentration was selected positively in the Acolman population (Table 1).

Univariate association between traits and fitness (selection differentials) indicated strong geographic variation in the pattern of selection acting on defensive traits. Secondary metabolites were more responsive than physical defenses in the presence of natural herbivores. Tropane alkaloids had positive, negative or neutral effects on fitness. Atropine concentration was selected against in three populations (San Martín, Santo Domingo and Valsequillo), and positively selected in one population (Acolman) (Table 2). Scopolamine concentration was positively selected in one population (Santo Domingo) and negatively selected in another population (Patria Nueva). Trichome density was negatively selected in one population only (Tzin Tzun Tzan) (Table 2). In two populations (Joquicingo and Sanabria) no evidence was detected of selection on plant defensive traits (Table 2).

**Table 2:** Multiple regression analyses to estimate linear selection gradients (*β*) for trichome density, atropine, and scopolamine concentration in eight populations of *Datura stramonium*.

Population	Main herbivore	Defensive trait	*β*	SE	*t*	*P*
Acolman (N = 31)	*Lema daturaphila*	Trichome density	−0.014	0.174	−0.08	0.937
		Atropine	**−0.985**	**0.293**	**−3.359**	**0.002**
		Scopolamine	**0.703**	**0.29**	**2.422**	**0.022**
Patria Nueva (N = 30)	*Lema daturaphila*	Trichome density	−0.096	0.124	−0.778	0.444
		Atropine	−0.068	0.13	−0.526	0.603
		Scopolamine	−0.178	0.153	−1.163	0.255
Joquicingo (N = 31)	*Lema daturaphila*	Trichome density	−0.194	0.196	−0.992	0.33
		Atropine	−0.258	0.196	−1.318	0.199
		Scopolamine	0.038	0.197	0.19	0.851
San Martín (N = 29)	*Lema daturaphila*	Trichome density	**0.328**	**0.146**	**2.244**	**0.034**
		Atropine	**−0.522**	**0.171**	**−3.054**	**0.005**
		Scopolamine	0.291	0.178	1.631	0.115
Tzin Tzun Tzan (N = 30)	*Epitrix parvula*	Trichome density	**−0.316**	**0.115**	**−2.759**	**0.01**
		Atropine	−0.208	0.14	−1.49	0.149
		Scopolamine	0.253	0.132	1.908	0.068
	*Epitrix parvula*					
Valsequillo (N = 33)		Trichome density	−0.009	0.089	−0.104	0.917
		Atropine	**−0.357**	**0.113**	**−3.154**	**0.003**
		Scopolamine	0.166	0.113	1.47	0.152
Sanabria (N = 34)	*Sphenarium purpurascens*	Trichome density	−0.189	0.2	−0.945	0.353
		Atropine	−0.335	0.222	−1.507	0.143
		Scopolamine	0.17	0.231	0.739	0.466
						
Santo Domingo (N = 30)	*Sphenarium purpurascens*	Trichome density	**0.04**	**0.014**	**2.769**	**0.01**
		Atropine	−0.012	0.014	−0.82	0.419
		Scopolamine	−0.018	0.013	−1.374	0.181

Significant gradients appear in bold-type fonts. Standard error (*SE*) of estimates, *t*-test value, and probability (*P*) are provided.

### Differential selection among herbivore species

**Figure 2:**
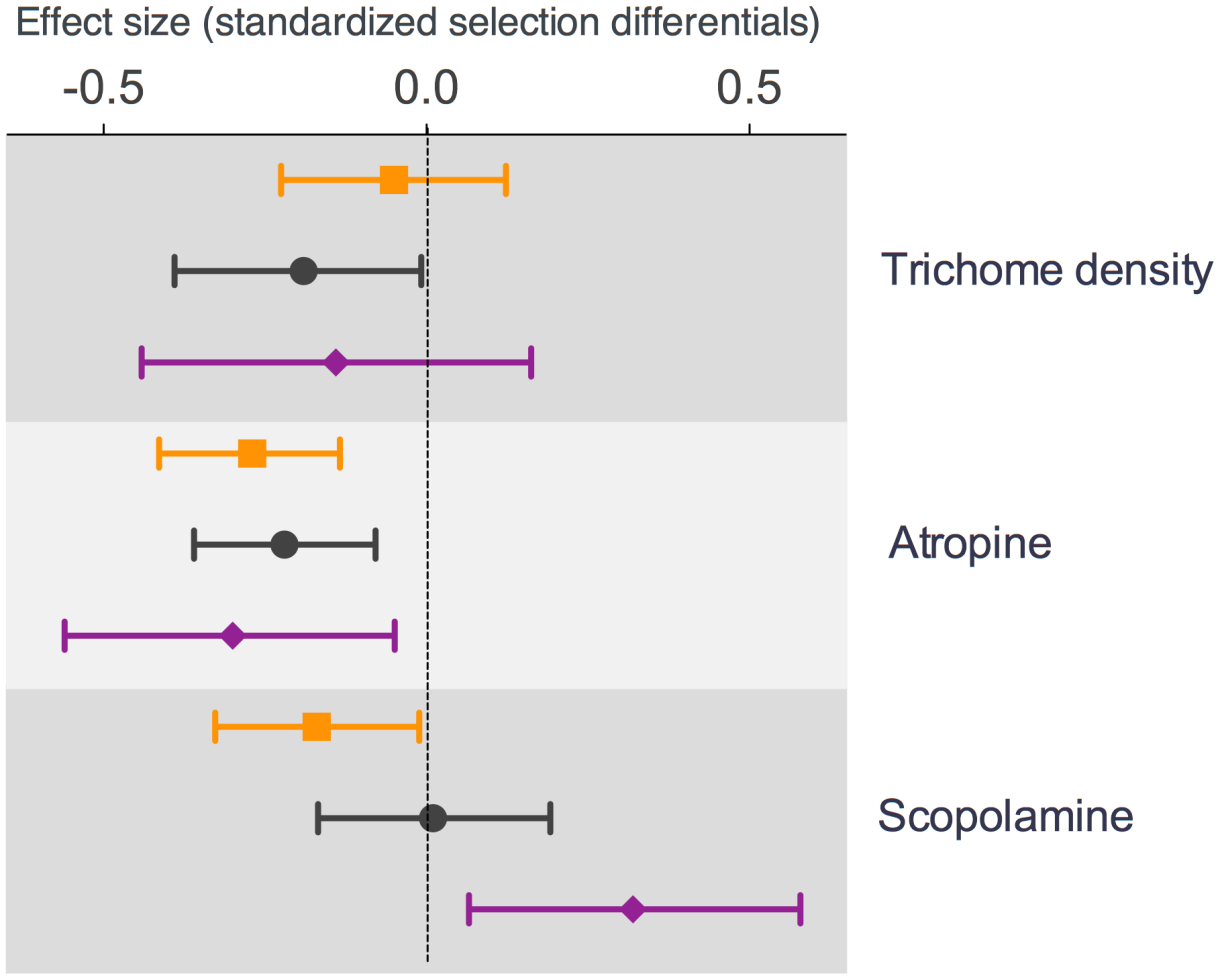
Forest plot showing the mean effect size for standardized selection differentials (*S*) of each defensive trait and corresponding confidence interval at 95%. Different colors and forms denote different species of herbivores: Tangerine squares: *Lema daturaphila*; black circles: *Epitrix parvula*, and purple diamonds: *Sphenarium purpurascens*. Corresponding values are reported in Table S3.

Trichome density was selected against in those populations eaten by *E. parvula*, while no consistent pattern was detected for the other herbivore species (Table S3). A comparison of selection differentials among herbivore species indicated a consistent trend in all species to select against the production of atropine, since mean effect sizes did not overlap zero and were negative (Figure 2). Mean effect sizes for scopolamine also showed a trend indicating that this alkaloid was selected against by the more specialized herbivore (*L. daturaphila*) followed by the less specialized beetle (*E. parvula*), and it was favored by the generalist grasshopper (Figure 2, Table S3). Mean effect sizes for scopolamine also showed a trend indicating that this alkaloid was selected against by the more specialized herbivore (*L. daturaphila*) followed by the less specialized beetle (*E. parvula*), while it was favored by the generalist grasshopper (Figure 2, Table S3). Differences between the mean effect sizes of selection differentials and those estimated for gradients of selection indicate that indirect selection is also consistent at this level of analysis. However, the contrast between the mean effect sizes (from differentials and gradients) indicates that direct selection is acting in a dominant fashion against scopolamine concentration (Table S3).

## Discussion

Our findings revealed significant geographic variation in selection patterns on defensive traits of *D. stramonium* in central Mexico. Despite this spatial variation, we were able to detect herbivore-specific effects on selection in plant defenses. All herbivore species selected for a reduction in the concentration of the “older” tropane alkaloid (atropine) suggesting that this secondary compound is no longer beneficial as a deterrent against herbivory, and that it entails a fitness cost to the host plant. In addition, the more toxic derived alkaloid (scopolamine) was more effective against the generalist rather than the specialist herbivores, which supports our initial expectation. Although previous studies in natural populations of *D. stramonium* showed significant selection favoring higher levels of trichome density [26], the present analyses detected a marginal fitness effect of trichome density in almost all populations examined. Overall the strong pattern of selection against the production of atropine is consistent with the previous finding of a positive geographic association between atropine and leaf damage within the same region [32].

Empirical evidence suggests that the spatial variation of traits that mediate the plant-herbivore interaction is a common phenomenon in nature. Yet understanding the origin and maintenance of such variation has proven to be challenging. Selection by herbivores is a major force shaping the evolution of plant defensive traits such as trichomes or secondary metabolites [5], [6]. However, along their distribution, plant species are exposed to specialized and/or generalist herbivores [11], [12]. Depending of their level of specialization, herbivores are expected to exert contrasting selective pressures on plant defense [11]. This is likely to produce spatially variable selection on defensive traits along the distribution of a species [49]. According the Geographic Mosaic Theory of Coevolution (GMCT, Thompson, [13]), selection mosaics constitute the raw material that promotes and maintains variation in those traits that are involved in species interactions. Here, we found evidence of spatially variable selection exerted by herbivores in both the chemical and physical defense of *D. stramonium*. Furthermore, in line with GMCT predictions, we found ample geographic variation in the defensive traits, similarly to what has been previously reported [26], [32] for trichome density, atropine and scopolamine concentrations of *D. stramonium*.

Estimation and interpretation of selection patterns is fundamental to form predictions about the evolution of defensive traits [50]. Differences in selective patterns among populations can lead to among-population differences in defensive traits [49]. In this study we found evidence of spatially variable phenotypic selection on defensive traits in populations facing different herbivore species (putative selective agents). Selection differentials indicate that both atropine and scopolamine were selected against in populations consumed by *L. daturaphila* and/or *E. parvula*. Thus, in the presence of genetic variation underlying the expression of tropane alkaloids, we suggest that atropine and scopolamine concentrations should be reduced in these populations. The contrast between differentials and gradients of selection for the studied alkaloids indicates that, while direct selection reduces atropine concentration, indirect selection reduces scopolamine. Since atropine is the precursor of scopolamine [51] and their concentrations are positively correlated (as detected in this study; see also Shonle & Bergelson, [16]), direct selection acting on atropine is likely conditioning the adaptive value of scopolamine as well. Nevertheless, in one of the studied populations (Santo Domingo), consumed by the generalist grasshopper *S. purpurascens*, selection favored an increase in scopolamine and a reduction in atropine concentrations. Although in this population no evidence of a positive correlation between alkaloids was detected, this contrasting selection could still explain why these costly chemical defenses are maintained. In addition, differences between selection differentials and gradients for trichome density and scopolamine concentration suggest that indirect selection represents an important force driving the evolution of plant chemical defense.

The specialist-generalist paradigm of host plant use by herbivores predicts that specialized herbivores should be less affected by plant defenses than generalists [52]. This expectation is based on the existence of a trade-off, such that being able to consume a diverse diet constrains the opportunities to specialize on a given host [53]–[55]. In turn, if plants are involved in a coevolutionary arms-race with herbivores through chemical defenses [56], recently evolved secondary plant compounds should be more effective against consumers than their ancestors [36]. Our results provide correlative evidence in support of theoretical expectations, since the precursor tropane alkaloid atropine had no positive effect on plant fitness, while the more derived alkaloid (scopolamine) was still effective against the generalist but not to the specialist herbivore (*L. daturaphila*). Accordingly, specialized herbivores may be even using atropine in order to select *D. stramonium* plants. Nonetheless, a pattern of directional selection for an increase in scopolamine was detected in one of the populations consumed by the generalist grasshopper, so it not possible to draw conclusions about the potential of this herbivore to affect the evolution of this tropane alkaloid. In addition, in one of the populations dominated by the specialized beetle *L. daturaphila*, atropine was favored by selection suggesting that the current coevolutionary state of the interaction at each population, together with gene flow among populations, could also account for the maintenance of these secondary compounds. Overall, the strong directional selection found for all herbivore species against atropine may explain the positive correlation recorded between herbivory damage and atropine concentration for a set of 28 plant populations within the same studied region in central Mexico [32].

## Conclusion

Empirical evidence suggests that spatial variation of traits that mediate interactions is a common phenomenon in nature. However, understanding the origin and maintenance of such variation has proven to be challenging. In this study, we provide evidence of spatially variable selection exerted by herbivores on physical and chemical defensive traits of *D. stramonium*. Local selective pressures are likely to produce the observed divergence in defensive traits at a geographic scale. However, further studies are still needed that explicitly evaluate the role of selection by herbivores in shaping trait divergence. In addition, future research should evaluate whether local adaptation to specialist and generalist herbivores occurs in nature, and to what extent it is mediated by defensive traits. Such research would increase our understanding of the great variation in defensive trait diversity in the wild.

## Supporting Information

Figure S1 **Among-population variation in a) leaf trichome density, b) atropine concentration, and c) scopolamine concentration in eight populations of** ***Datura stramonium*** **in central Mexico.** Bars represent average value +1 standard error.
(DOC)

Table S1 ***Datura stramonium*** **populations sampled in August-September 2011.** DS  =  Desert shrub, POF  =  Pine-Oak forest TDF  =  Tropical deciduous forest.
(DOC)

Table S2 **Correlations (** ***r*** **) between trichome density, scopolamine, and atropine concentration in eight populations of** ***Datura stramonium*** **in central Mexico.** Significant correlations appear in bold-type fonts.
(DOC)

Table S3 **Effect sizes for selection differential (** ***S*** **) and gradients (** ***β*** **) of selection with their corresponding confidence intervals at 95% (in parentheses).** An omnibus test (*Q_m_*) evaluates whether parameters are equal among groups (*i.e., H_0_ = β_1_ = … = β_p_ = *0). *, *P*<0.05; ***, *P*<0.001; n. s., not significant. Confidence intervals at 95% in bold-type font do not overlap with zero value.
(DOC)
