# Correction: Selection Mosaic Exerted by Specialist and Generalist Herbivores on Chemical and Physical Defense of *Datura stramonium*

**DOI:** 10.1371/journal.pone.0145554

**Published:** 2015-12-16

**Authors:** Guillermo Castillo, Laura L. Cruz, Rosalinda Tapia-López, Erika Olmedo-Vicente, Diego Carmona, Ana Luisa Anaya-Lang, Juan Fornoni, Guadalupe Andraca-Gómez, Pedro L. Valverde, Juan Núñez-Farfán

The fourth author’s name is spelled incorrectly. The correct name is: Erika Olmedo-Vicente.
