# Supplementary figures and images for: Selection Mosaic Exerted by Specialist and Generalist Herbivores on Chemical and Physical Defense of Datura stramonium
Source: PLoS One. 2014 Jul 22;9(7):e102478. doi: 10.1371/journal.pone.0102478 (PMC4106780; doi:10.1371/journal.pone.0102478)

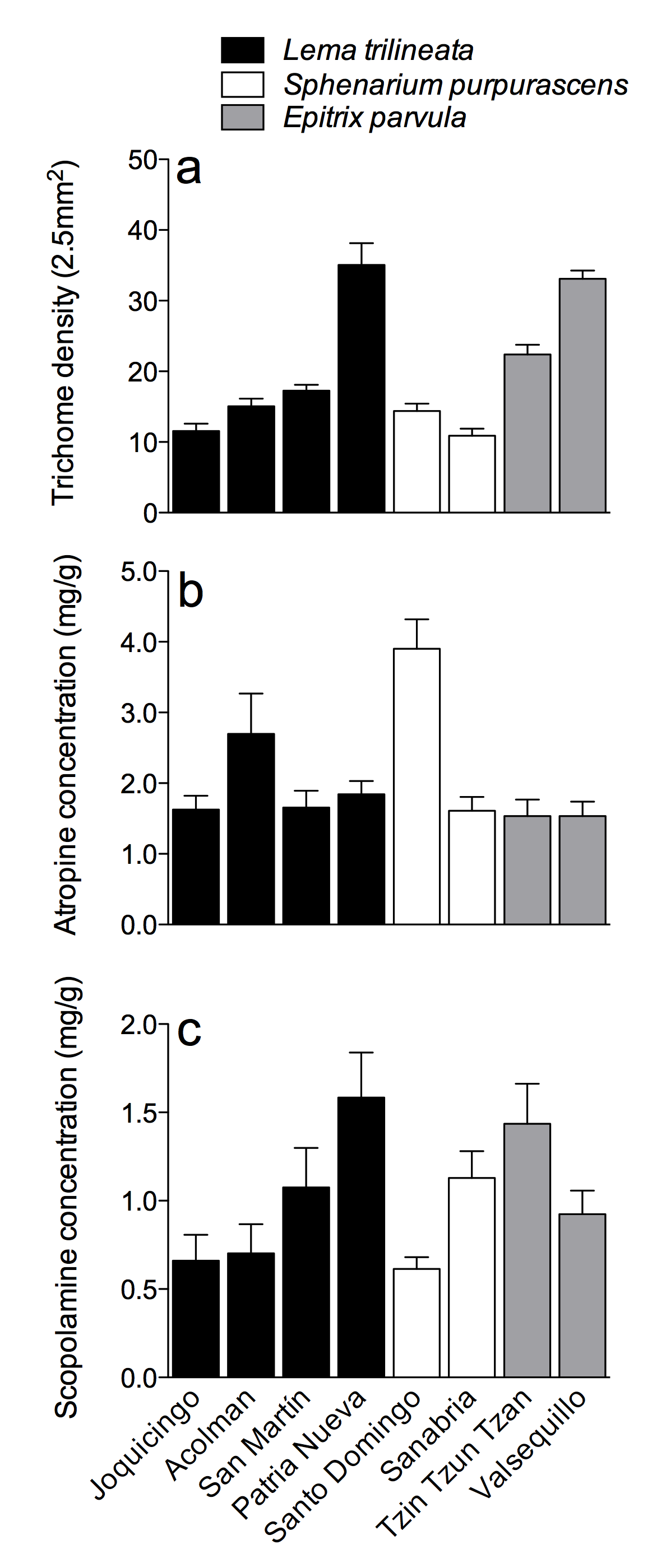

Supplement: Figure S1 — Among-population variation in a) leaf trichome density, b) atropine concentration, and c) scopolamine concentration in eight populations of Datura stramonium in central Mexico. Bars represent average value +1 standard error. (DOC) [file pone.0102478.s001.doc]
