# Supplementary material for: Selection Mosaic Exerted by Specialist and Generalist Herbivores on Chemical and Physical Defense of Datura stramonium
Source: PLoS One. 2014 Jul 22;9(7):e102478. doi: 10.1371/journal.pone.0102478 (PMC4106780; doi:10.1371/journal.pone.0102478)
Supplement: Table S1 — Datura stramonium populations sampled in August-September 2011. DS = Desert shrub, POF = Pine-Oak forest TDF = Tropical deciduous forest. (DOC) [file pone.0102478.s002.doc]

| **Population** | **State** | **Vegetation type** | **Main Herbivore** | **Latitude** | **Longitude** | **Altitude (m. a. s. l.)** | **Precipitation (mm^3^)** | **Temperature (°C)** |
| --- | --- | --- | --- | --- | --- | --- | --- | --- |
| Acolman | Mexico | DS | *L. daturaphila* | 19.6871 | -98.8352 | 2294 | 700 | 14.9 |
| Joquicingo | Mexico | POF | *L. daturaphila* | 19.1153 | -99.5181 | 2583 | 1000 | 14 |
| Patria Nueva | Hidalgo | DS | *L. daturaphila* | 20.3765 | -99.0481 | 1922 | 500 | 16.6 |
| San Martín | Puebla | TDF | *L. daturaphila* | 19.3048 | -98.4823 | 2327 | 800 | 15 |
| Tzin Tzun Tzan | Michoacán | POF | *E. parvula* | 19.6362 | -101.5924 | 2047 | 1000 | 16.4 |
| Valsequillo | Puebla | DS | *E. parvula* | 18.95 | -98.1803 | 2064 | 800 | 17.7 |
| Sanabria | Michoacán | POF | *S. purpurascens* | 19.577 | -101.57 | 2052 | 1000 | 16.4 |
| Santo Domingo | Morelos | TDF | *S. purpurascens* | 19.0118 | -99.0627 | 2068 | 1000 | 16.6 |
