# Supplementary material for: Selection Mosaic Exerted by Specialist and Generalist Herbivores on Chemical and Physical Defense of Datura stramonium
Source: PLoS One. 2014 Jul 22;9(7):e102478. doi: 10.1371/journal.pone.0102478 (PMC4106780; doi:10.1371/journal.pone.0102478)
Supplement: Table S2 — Correlations ( r ) between trichome density, scopolamine, and atropine concentration in eight populations of Datura stramonium in central Mexico. Significant correlations appear in bold-type fonts. (DOC) [file pone.0102478.s003.doc]

| **Population** | **Trait** | **by trait** | ***r*** | ***P*** |
| --- | --- | --- | --- | --- |
| Acolman | Atropine | Trichome density | -0.171 | 0.3557 |
|  | Scopolamine | Trichome density | -0.091 | 0.6234 |
|  | Scopolamine | Atropine | 0.805 | **<0.0001** |
|  |  |  |  |  |
| Joquicingo | Atropine | Trichome density | 0.237 | 0.2144 |
|  | Scopolamine | Trichome density | 0.261 | 0.1706 |
|  | Scopolamine | Atropine | 0.087 | 0.6537 |
|  |  |  |  |  |
| Patria Nueva | Atropine | Trichome density | 0.101 | 0.5929 |
|  | Scopolamine | Trichome density | 0.529 | **0.0026** |
|  | Scopolamine | Atropine | 0.59 | **0.0006** |
|  |  |  |  |  |
| San Martin | Atropine | Trichome density | -0.02 | 0.9127 |
|  | Scopolamine | Trichome density | -0.277 | 0.1373 |
|  | Scopolamine | Atropine | 0.58 | **0.0008** |
|  |  |  |  |  |
| Sanabria | Atropine | Trichome density | 0.185 | 0.3103 |
|  | Scopolamine | Trichome density | 0.365 | **0.0397** |
|  | Scopolamine | Atropine | 0.561 | **0.0006** |
|  |  |  |  |  |
| Santo Domingo | Atropine | Trichome density | 0.287 | 0.1306 |
|  | Scopolamine | Trichome density | 0.065 | 0.7363 |
|  | Scopolamine | Atropine | 0.056 | 0.7717 |
|  |  |  |  |  |
| Tzin Tzun Tzan | Atropine | Trichome density | 0.315 | 0.0785 |
|  | Scopolamine | Trichome density | 0.02 | 0.9127 |
|  | Scopolamine | Atropine | 0.527 | **0.0019** |
|  |  |  |  |  |
| Valsequillo | Atropine | Trichome density | 0.08 | 0.6566 |
|  | Scopolamine | Trichome density | -0.082 | 0.6478 |
|  | Scopolamine | Atropine | 0.61 | **0.0001** |
