# Supplementary material for: Selection Mosaic Exerted by Specialist and Generalist Herbivores on Chemical and Physical Defense of Datura stramonium
Source: PLoS One. 2014 Jul 22;9(7):e102478. doi: 10.1371/journal.pone.0102478 (PMC4106780; doi:10.1371/journal.pone.0102478)
Supplement: Table S3 — Effect sizes for selection differential ( S ) and gradients ( β ) of selection with their corresponding confidence intervals at 95% (in parentheses). An omnibus test (Qm) evaluates whether parameters are equal among groups (i.e., H0 = β1 = … = βp = 0). *, P<0.05; ***, P<0.001; n. s., not significant. Confidence intervals at 95% in bold-type font do not overlap with zero value. (DOC) [file pone.0102478.s004.doc]

|  | Trichome density | | Atropine | | Scopolamine | |
| --- | --- | --- | --- | --- | --- | --- |
|  | *S* | *β* | *S* | *β* | *S* | *β* |
| *d. f . =* 3 | *Q_m_* = 5.32 n. s. | *Q_m_* = 10.451* | *Q_m_* = 29.68*** | *Q_m_* = 24.63*** | *Q_m_*= 10.49* | *Q_m_* = 8.371* |
| *L. daturaphila* | -0.05 | 0.02 | **-0.27** | **-0.31** | **-0.17** | 0.09 |
|  | (-0.225, 0.123) | (-0.129, 0.171) | **(-0.414, -0.134)** | **(-0.478, -0.135)** | **(-0.327, -0.011)** | (-0.088, 0.282) |
| *E. parvula* | **-0.19** | -0.12 | **-0.22** | **-0.29** | 0.01 | **0.2** |
|  | **(-0.390, -0.008)** | (-0.264, 0.013) | **(-0.360, -0.079)** | **(-0.470, -0.126)** | (-0.168, 0.192) | **(0.034, 0.371)** |
| *S. purpurascens* | -0.14 | **0.04** | **-0.3** | -0.01 | **0.32** | -0.02 |
|  | (-0.441, 0.162) | **(0.010, 0.067)** | **(-0.560, -0.049)** | (-0.041, 0.015) | **(0.066, 0.579)** | (-0.044, 0.008) |
